# Computational studies on defect chemistry and Li-ion conductivity of spinel-type LiAl_5_O_8_ as coating material for Li-metal electrode

**DOI:** 10.1038/s41598-022-20289-2

**Published:** 2022-10-05

**Authors:** Shuntaro Miyakawa, Shogo Matsuda, Naoto Tanibata, Hayami Takeda, Masanobu Nakayama, Takaya Saito, Svetlana Fukuchi

**Affiliations:** 1 Advanced Battery Research Office, Research Institute of Advanced Technology, SoftBank Corporation, Kaigan, Minato-Ku, Tokyo, 105-7529 Japan; 2 Department of Advanced Ceramics, Nagoya Institute of Technology, Gokiso, Showa-ku, Nagoya, Aichi 466-8555 Japan

**Keywords:** Materials for energy and catalysis, Theory and computation

## Abstract

Li-metal rechargeable batteries are an attractive option for devices that require an extremely high specific energy density, high robustness, and long-term durability, such as high-altitude platform stations. However, Li dendrite growth during charge–discharge cycling causes short-circuit problems. One technical solution is to form an intermediate layer between the Li metal and electrolyte. This interfacial layer should possess mechanical strength, electrochemical stability in the presence of Li, and Li-ion conductivity. In this study, the Li-ion conductivity of spinel-type LiAl_5_O_8_ was investigated using first-principles density functional theory and force field molecular dynamics calculations. The calculation results confirmed that stoichiometric LiAl_5_O_8_ compounds do not exhibit Li-ion conductivity, whereas off-stoichiometric compounds with excess Li show long-range Li-ion diffusion. The evaluated activation energy was 0.28 eV, which is as low as that of well-known fast Li-ion conductors, such as garnet-type Li_7_La_3_Zr_2_O_12_. However, the extrapolated Li-ion conductivity at 298 K was relatively low (~ 10^−6^ S/cm) owing to the limited formation of migration pathways.

## Introduction

Full-scale use of fifth-generation (5G) cellular network technologies began in 2020; however, wireless communication signals have not reached several regions in the world, and there is a growing disparity between the areas with and without advanced digitization. In response to this issue, we propose that a society without digital disparity could be created by providing a stratospheric communication platform based on high-altitude platform stations (HAPSs)^1^. HAPSs are unmanned gliders that fly in the stratosphere to provide communication signals over a wide area. These devices are battery powered and utilize solar energy to recharge. However, the battery accounts for the largest proportion of weight among the airframe components. Batteries with a specific energy of 400 Wh/kg or more are required to meet a minimum flight period (12 h). This target is not possible to meet using commercial Li-ion batteries, which have a specific energy of approximately 300 Wh/kg^2^. This makes it difficult to meet the weight requirements of practical HAPS airframes.

The Li metal, which has a theoretical capacity of 3680 Ah/kg, has been proposed as an anode material to increase the specific energy of Li-ion batteries. Li metal has the potential to considerably exceed HAPS development goals, making it a promising next-generation anode material. However, Li-metal anodes experience dendrite growth over a number of charge–discharge cycles, which eventually breaks through the separator and causes internal short circuits^3,4^. Several approaches have been proposed to solve this problem, including (1) improving the separator properties, such as pore arrangement and coating layer formation^5,6^, (2) forming an artificial intermediate layer (solid electrolyte interphase (SEI)) between the Li-metal anode and electrolyte^7^, (3) combining nanostructured materials and Li metal to form a composite anode^8,9^, and (4) achieving stable SEI formation by tailoring the electrolyte composition^10,11^. Although these proposed approaches can effectively solve the problem arising from the dendritic growth of Li metal during charge–discharge, further improvements are required to satisfy the reversibility, durability, and rate performance for effective realization. Among the above-mentioned approaches, formation of an artificial SEI is the most attractive option. For example, Li et al.^12^ reported that an ultrathin Li_3_PO_4_ layer (approximately 200 nm thick) was formed on a Li-metal anode through an in-situ reaction. The Li||LiFePO_4_ battery showed stable performance over 200 charge–discharge cycles at a rate of 0.5 °C^13^. Similarly, Chen et al.^14^ deposited LiF on a Li-metal anode using atomic layer deposition. The cell maintained a Coulombic efficiency of > 99.5% over 170 charge–discharge cycles^14^. Various other candidate materials for artificial SEI have also been reported, including organic compounds^15,16^, Li alloys^17,18^, and organic–inorganic hybrid materials^19,20^.

We recently confirmed that inserting an ultrathin Al_2_O_3_ layer as an artificial SEI suppresses the side reactions between the Li metal and electrolyte. The cell maintained a low overvoltage even after 500 h of continuous operation at a rate of > 1 mA/cm^2^^13^. The minimum requirements for artificial SEIs include electronic insulation, (electro)chemical stability, stability in the presence of Li metal, and sufficient Li-ion conductivity^21^. Although Al_2_O_3_ meets most of these requirements, it is thermodynamically unstable in the presence of Li metal. Several conceivable decomposition products are present between Li and Al_2_O_3_, including LiAl_5_O_8_, Li_5_AlO_4_, and LiAlO_2_, in the Li–Al–O ternary system^22^. Among them, according to previous reports, LiAl_5_O_8_ is formed according to the following reaction^23–25^.

$$ 8{\text{Al}}_{2} {\text{O}}_{3} + 3{\text{Li}} \to 3{\text{LiAl}}_{5} {\text{O}}_{8} + {\text{Al}}. $$

Because decomposition of the artificial SEI during cycling presumably causes variations in the current density, the materials used for artificial SEI formation should be resistant to decomposition. Therefore, pre-coating with spinel-type LiAl_5_O_8_ may improve the durability of the artificial SEI and the stability of Li-metal batteries and prevent the decomposition reaction between Al_2_O_3_ and Li metal. In addition, because LiAl_5_O_8_ contains Li, it is expected to exhibit Li-ion conductivity. Recently, Mo et al.^23^ systematically evaluated the potential window, defect structure, and Li-ion conductivity of LiAl_5_O_8_ using first-principles calculations^22^. They found that LiAl_5_O_8_ has a relatively large potential window and low Li-ion migration energy (0.33 eV), confirming that LiAl_5_O_8_ is a promising material for artificial SEI formation. In this study, we aimed to elucidate the diffusion mechanism of Li ions in LiAl_5_O_8_ and quantify the diffusion coefficient via multiscale calculation combining first-principles calculations and force field molecular dynamics (FFMD) simulations, with consideration of the influence of the defect arrangement.

## Methods

The optimal structure and corresponding total electronic energy of LiAl_5_O_8_ and its defective relatives were evaluated using first-principles density functional theory (DFT) calculations. The plane-wave basis and projector-augmented wave methods^26^ were implemented in the Vienna ab initio simulation package (VASP)^27,28^. In addition, the Perdew–Burke–Ernzerhof generalized gradient approximation for solids (GGA-PBEsol)^29,30^ was applied to approximate electron–electron interactions.

To evaluate the defect formation energy of multiple point defect models included in a system, the arrangement of the point defects must be optimized. In this study, a stable defect arrangement was determined using a genetic algorithm (GA)^31,32^. The lattice used was a conventional spinel lattice (M_24_O_32_) modeled with octahedral vacancy sites. Because GAs generate several thousand possible stable defect arrangements, even for a single composition, a plane-wave cutoff energy of 380 eV and k-point mesh of 1 × 1 × 1 were applied to reduce the computational cost, after which five energetically stable candidate structures were extracted. For these candidate structures, a plane-wave cutoff energy of 500 eV and k-point mesh of 3 × 3 × 3 were applied, and structure relaxation calculations were performed again; the most stable structure was then selected as the final structure.

The ionic conductivities of LiAl_5_O_8_ and related materials were evaluated using first-principles molecular dynamics (FPMD) calculations^33,34^. FPMD calculations were performed at 1273 K for 100 ps. The conditions for the FPMD calculations were the same as those for the structure relaxation calculations, with minor modifications to the cutoff energy and k-point division to reduce the computational cost while maintaining calculation accuracy. Specifically, the cutoff energy of the planar basis function was set to 350 eV and the k-point division was accepted as 1 × 1 × 1 (only the Γ point). Molecular dynamics (MD) calculations were performed using a Nosé–Hoover thermostat and the NVT canonical ensemble. The MD step interval was 1 fs. After performing the FPMD calculations, the mean square displacement (MSD) was calculated for each element.

From the viewpoint of computational cost, evaluations of Li-ion migration by FPMD are limited to relatively small systems (small numbers of atoms and low simulation times). Thus, unreasonably high-temperature simulations are often performed to obtain sufficient statistical data for the site-to-site hopping events of Li ions. In this study, we optimized the FFMD parameters through a cuckoo search^35–37^ to reproduce the crystal structure data for the FPMD calculations (i.e., radial distribution function (RDF), angular distribution function (ADF), and lattice volume) with a relatively low simulation time (2 ps) using the NPT ensemble (invariant particle number (*N*), constant pressure (1 atm) (*P*), and constant temperature (*T*)^37,38^). The force field (FF) parameters and MD calculations were optimized using the NAP software package^37,39^. A high-throughput FF function was used, namely, the bond valence FF function proposed by Adams et al.^40,41^, to which the Stillinger–Weber-type angular (three-body) potential^42^ was added. Then, using the optimized FF parameters, FFMD calculations were performed with the NVT ensemble (invariant particle number (*N*), constant volume (*V*), and constant temperature (*T*)), and the trajectories of the ions were recorded.

## Structure description

**Figure 1:**
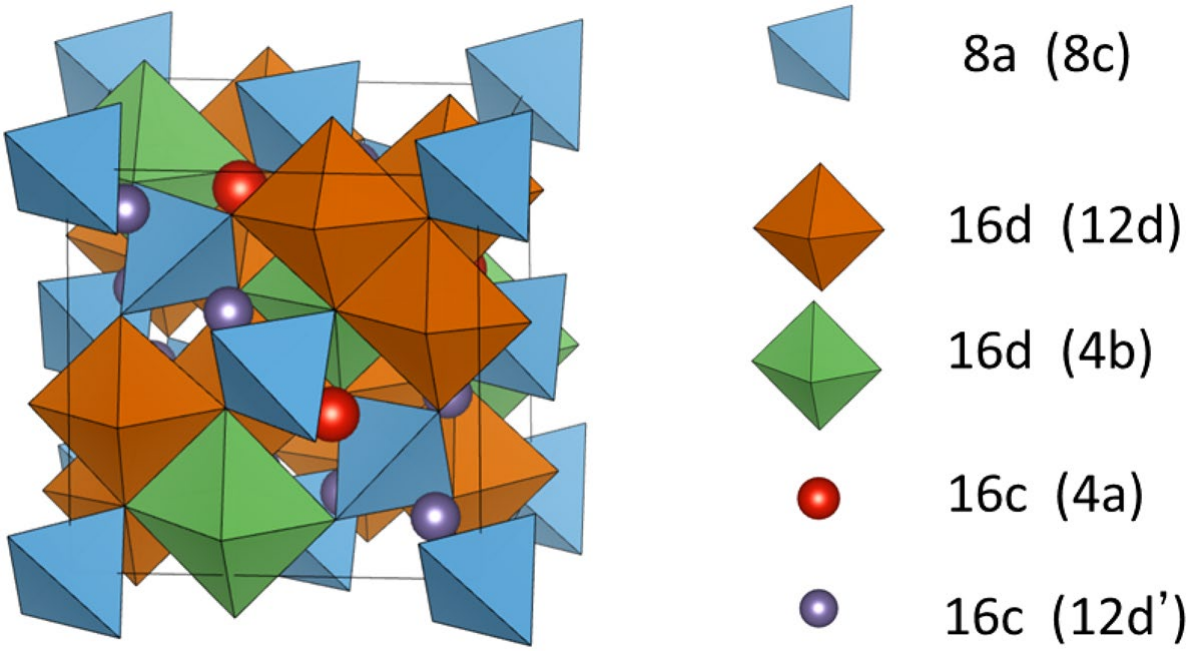
Schematic representation of the spinel-type crystal structure. Wyckoff positions of the sites occupied by cations and vacancies with maximum oxygen packing are described on the right. Wyckoff positions in the *Fd*$\overline{3 }$*m* and *P*4_3_32 space groups are written without and with brackets, respectively.

Figure 1 shows the conventional spinel-type crystal structure of M_3_O_4_ (M = metal). The spinel-type structure belongs to space group *Fd*$\overline{3 }$*m* and has a cubic close-packed (ccp) configuration. In the ccp structure, oxide ions occupy the 32e sites and metal ions occupy one-eighth of the tetrahedral sites (8a) and half the octahedral sites (16d). In electrode materials such as LiMn_2_O_4_ and Li_4_Ti_5_O_12_, Li ions occupy the 8a sites and traverse the diffusion pathway via the octahedral 16c site vacancies, which are at the midpoints of the 8a sites. In contrast, the metal–oxygen array in LiAl_5_O_8_ is equivalent to the spinel structure, where Li ions occupy octahedral sites and Al ions occupy tetrahedral and octahedral sites^43^. An ordered array is formed with the octahedral sites occupied by both types of cations, Li and Al, which corresponds to space group *P*4_3_32. The tetrahedral sites, which are occupied by Al ions, correspond to 8c sites. The octahedral sites are divided into 4b sites, which are mainly occupied by Li, and 12d sites, which are occupied by Al. The 16c octahedral vacancy sites in space group *Fd*$\overline{3 }$*m* correspond to sites 4a and 12d in space group *P*4_3_32. Because the same Wycoff symbol is used for the 12d cation-occupied sites and the 12d vacancy sites, herein, unless otherwise noted, the vacancy sites are written as 12d′ to distinguish them.

## Results and discussion

### Defect model

**Figure 2:**
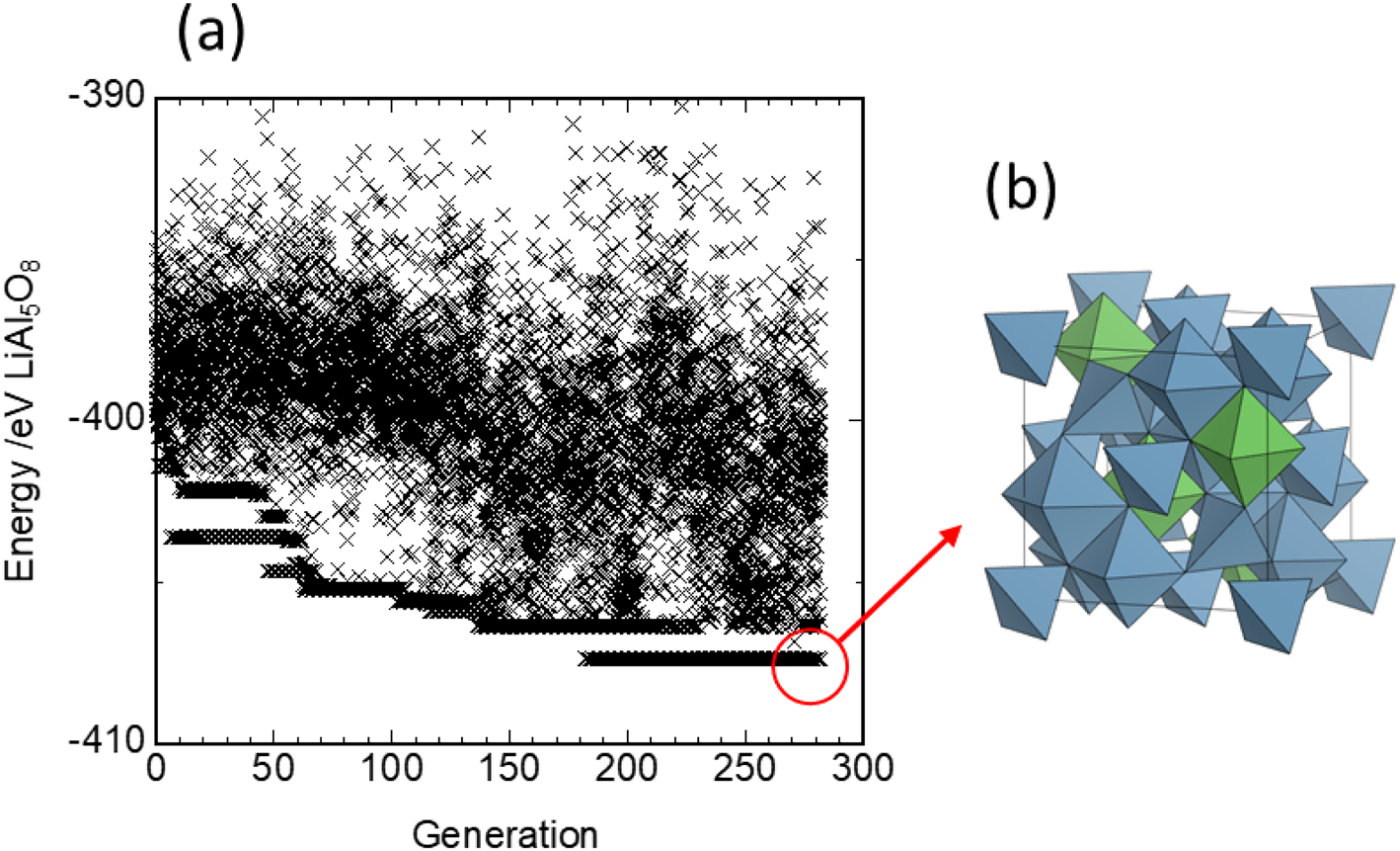
Determination of the most stable Li/Al/vacancy arrays of defect-free LiAl_5_O_8_ (Model 1) using a genetic algorithm (GA). (**a**) Total electronic energy of GA-generated individual structures as a function of generation for spinel-type LiAl_5_O_8_. One generation consists of 20 individuals. (**b**) Most stable structure among GA-generated structure candidates. The centers of the blue and green polyhedra are occupied by Al and Li ions, respectively.

Mo et al.^23^ systematically evaluated the potential window, defect structure, and Li-ion conductivity of LiAl_5_O_8_ using first-principles calculations. They considered the defects to be dilute and therefore calculated the defect energy without considering defect–defect interactions. In the present study, four structural models (denoted as Models 1–4) with neutral lattice charges and optimized defect arrays were investigated, with reference to the work of Mo et al.^23^. Model 1 is a perfect (defect-free) crystal of LiAl_5_O_8_^22^. In actual crystals, the tetrahedral 8a sites and octahedral 16d and 16c sites in the conventional spinel structure (space group *Fd*$\overline{3 }$*m*) can be occupied by Li, Al, and vacancies, resulting in 4 × 10^16^ possible arrays. A GA was used to efficiently determine the most stable Li/Al/vacancy arrays. Figure 2a shows the search process using the GA. Convergence to the most stable energy state occurred in approximately 180 generations (20 individuals per generation). Figure 2b shows the final structure. The cations were distributed only at the 8a and 16d sites, and all tetrahedral 8a sites were occupied by Al. These results are in good agreement with experimental reports^43^. Furthermore, the octahedra occupied by Li ions were isolated and not adjacent. Thus, the most stable structure of Model 1 was the *P*4_3_32-type structure, as displayed in Fig. 1.

For Models 2–4, we considered structures containing Li vacancies or interstitial Li point defects, which are assumed to contribute to Li-ion diffusion. Model 2 contained excess Al_2_O_3_, which generated Li vacancies according to reaction (1). The reactions are shown using Kröger–Vink notation.

1 $$2{\mathrm{Al}}_{2}{\mathrm{O}}_{3}\to {\mathrm{Al}}_{\mathrm{Li}}^{\cdot \cdot }+2{\mathrm{V}}_{\mathrm{Li}}^{\mathrm{^{\prime}}}+15{\mathrm{Al}}_{\mathrm{Al}}^{\times }+24{\mathrm{O}}_{\mathrm{O}}^{\times }.$$

Model 3 contained Schottky-type defects, in which both O and Li vacancies were generated:

2 $$2{\mathrm{LiAl}}_{5}{\mathrm{O}}_{8}\to {\mathrm{ V}}_{\mathrm{O}}^{\cdot \cdot }+{2\mathrm{V}}_{\mathrm{Li}}^{\mathrm{^{\prime}}}+{\mathrm{Al}}_{\mathrm{Al}}^{\times }+15{\mathrm{O}}_{\mathrm{O}}^{\times }+{\mathrm{Li}}_{2}\mathrm{O}.$$

Finally, Model 4 contained excess Li_2_O, which generated interstitial Li point defects:

3 $$8{\mathrm{Li}}_{2}\mathrm{O}\to {15\mathrm{Li}}_{\mathrm{i}}^{\cdot }+{5\mathrm{V}}_{\mathrm{Al}}^{\mathrm{^{\prime}}\mathrm{^{\prime}}\mathrm{^{\prime}}}+{\mathrm{Li}}_{\mathrm{Li}}^{\times }+8{\mathrm{O}}_{\mathrm{O}}^{\times }.$$

For Models 2–4, we considered cells that were twice the size of conventional spinel lattice cells (M_24_O_32_). The compositions of Models 2, 3, and 4 were set to Li_5_Al_41_O_64_, Li_6_Al_40_O_63_, and Li_11_Al_39_O_64_, respectively. Similar to Model 1, the cation and anion arrays were optimized by the GA, and the total electronic energies of the structures were evaluated.

Using the obtained energy values for each structural model, the defect generation energies for Models 2–4 were determined; these are summarized in Table 1. Assuming a synthesis temperature of 1273 K, the Li vacancy or interstitial Li defect concentration was evaluated as follows:

4 $$K=\mathrm{exp}\left(-\frac{{E}_{\mathrm{def}}}{{k}_{\mathrm{B}}T}\right),$$

where *K* is the equilibrium constant based on Eqs. (1)–(3), and *E*_def_, *k*_B_, and *T* are the defect formation energy, Boltzmann constant, and absolute temperature, respectively.

**Table 1:** Defect generation energies and defect concentrations at 1273 K for Models 2–4 evaluated using first-principles calculations.

Model ID	Composition	Defect species (carrier)	Defect concentration at 1273 K
Model 2	Li_5_Al_41_O_64_	${\mathrm{V}}_{\mathrm{Li}}^{^{\prime}}$	7.8 × 10^−3^
Model 3	Li_6_Al_40_O_63_	${\mathrm{V}}_{\mathrm{Li}}^{^{\prime}}$	4.4 × 10^−9^
Model 4	Li_11_Al_39_O_64_	${\mathrm{Li}}_{\mathrm{i}}^{^{\prime}}$	9.8 × 10^−2^

Table 1 lists the calculated defect concentrations for Models 2–4 at 1273 K. The results show that Model 4, in which interstitial Li is generated, has the highest defect concentration. The results show that Model 4 is the most stable, which is consistent with the results of Mo et al.^23^. In the GA-optimized structure of Model 4, the cations no longer occupy the tetrahedral 8a sites; instead, all cations occupy the octahedral 16c and 16d sites, resulting in a rock-salt-type structure. These results are consistent with previously obtained results indicating that the introduction of small amounts of Mg into a spinel-type structure causes a transition to a rock-salt type structure^44^.

### Diffusivity of Li ions in solids

**Figure 3:**
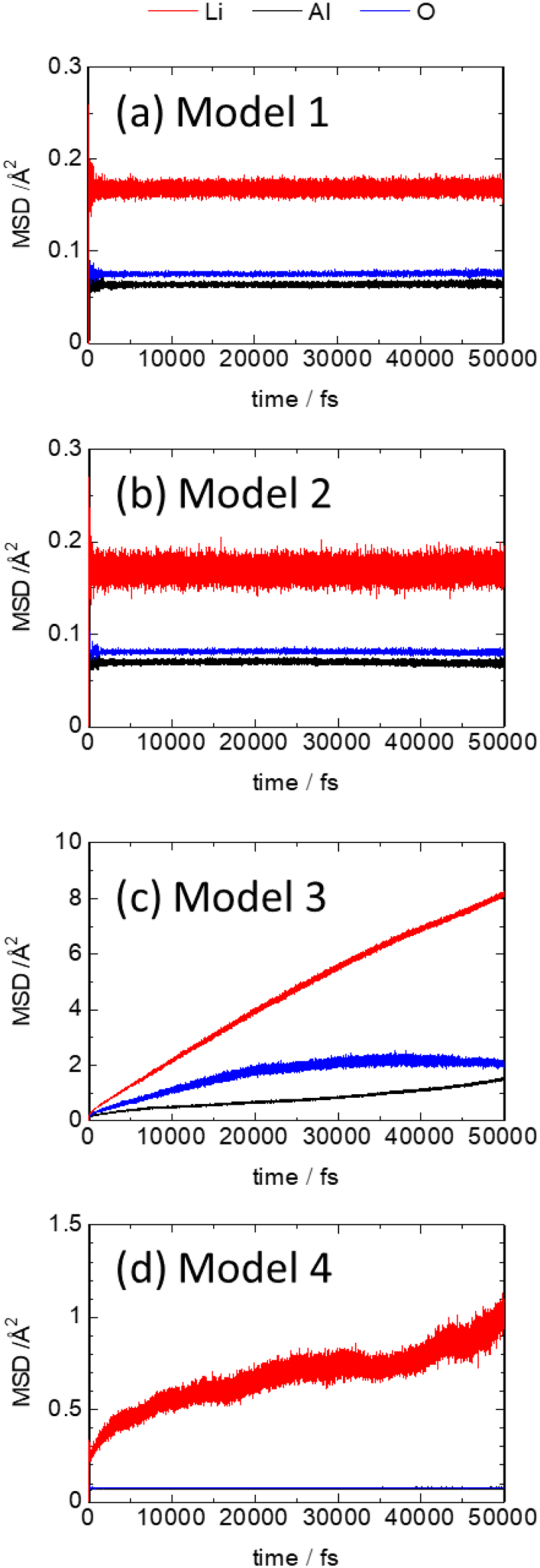
Mean square displacement (MSD) plots of Li, Al, and O obtained by first-principles molecular dynamics (FPMD) calculations for Models 1–4 at 1273 K. (**a**) Model 1, (**b**) Model 2, (**c**) Model 3, and (**d**) Model 4.

The FPMD calculations were performed for √2 × √2 × 1 supercells of the GA-optimized structures of Models 1–4 for temperatures ranging from 298 to 1273 K with sampling over 60,000 steps (60 ps). The MSD of the constituent ions was evaluated. Figure 3 shows the MSD at 1273 K as a function of simulation time. In Models 1, 2, and 4, the MSDs of Al and O were less than 1 Å^2^ at all temperatures and compositions, and no displacement, except for the thermal vibration effect, was observed. In contrast, in Model 3, the MSD changed over time for all ionic species (Li, Al, and O), suggesting that the spinel structure decomposes over time; therefore, we excluded this model for further consideration. There was no marked increase in the MSD of Li over time in Models 1 and 2, and only thermal vibration effects were observed. In contrast, the MSD of Li increased over time in Model 4, indicating that Li-ion diffusion occurred via the hopping mechanism. Therefore, the contribution of Li vacancies to diffusion is small, and conduction is accelerated by Li ions at interstitial sites. The diffusion coefficient (*D*) of Li^44^ at 1273 K was determined to be 2 × 10^−7^ cm^2^/s from the slope of the MSD profile. This diffusion coefficient is approximately two orders of magnitude lower than the FPMD-derived value^45^ for the well-known garnet-type solid electrolyte Li_7_La_3_Zr_2_O_12_ (1.3 × 10^−5^ cm^2^/s). However, the MSD is just over 1 Å^2^ for a simulation time of 50 ps (1273 K), suggesting that the number of Li-ion hopping events may not satisfy statistical requirements for the quantity required to estimate the diffusion coefficient. In addition, because the square root of the MSD is smaller than the lattice constant of any of the *a*, *b*, or *c* axes (~ 8 Å), it is not clear whether there are connected pathways to facilitate Li-ion diffusion throughout the lattice. Prolongation of the simulation time is required to verify the connectivity of the diffusion pathways, which is difficult from the viewpoint of computational cost.

**Figure 4:**
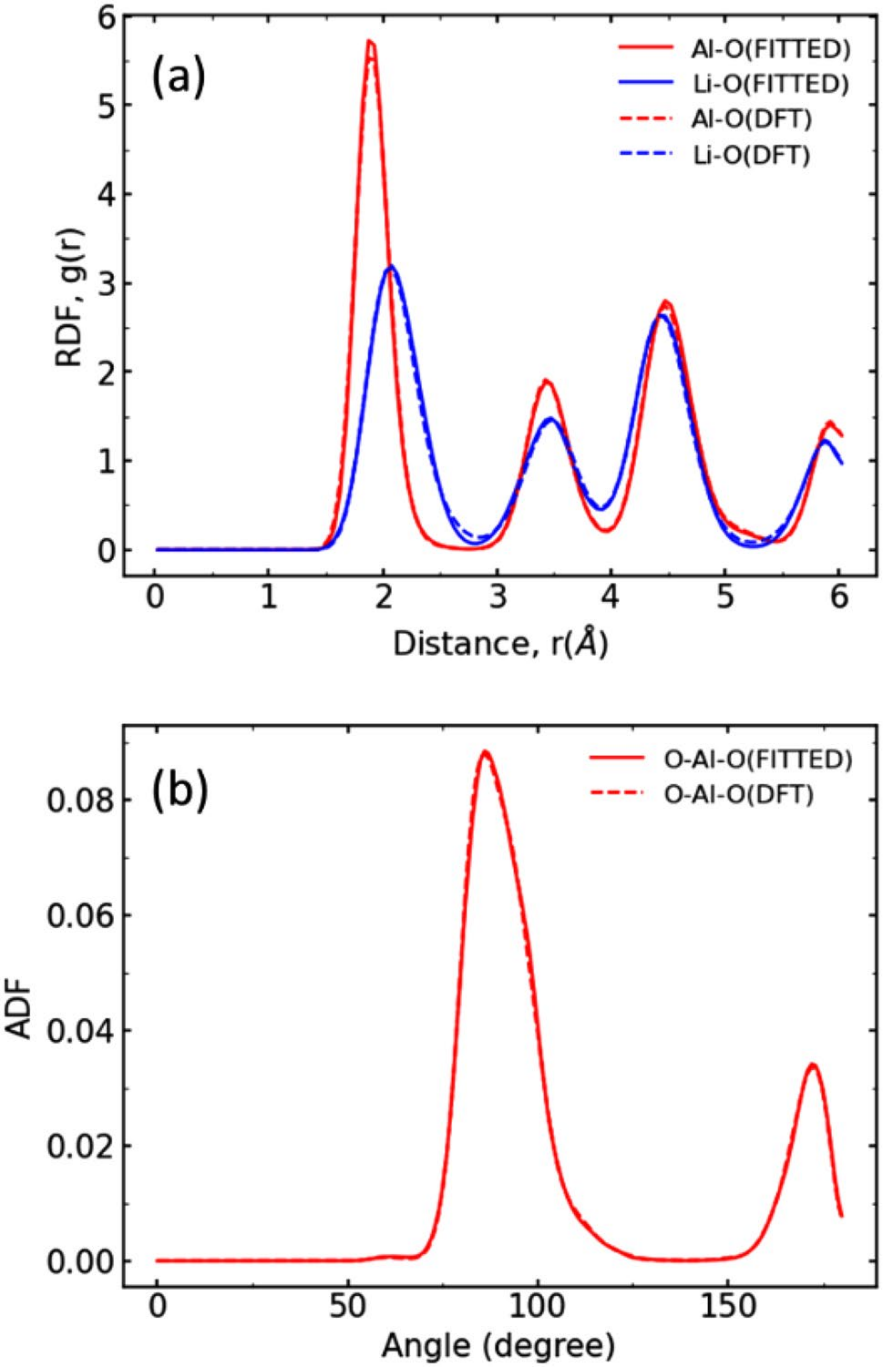
Comparison of structural data obtained by first-principles molecular dynamics (FPMD) and force field molecular dynamics (FFMD) with optimized force field parameters. (**a**) Radial distribution function (RDF) and (**b**) angular distribution function (ADF).

The MD calculations with high-throughput FF simulations were performed to quantitatively evaluate the diffusion coefficients. The FF parameters were determined by a cuckoo search to reproduce the structural data (RDF, ADF, and lattice volume) of the 2 ps FPMD calculations. This optimization was performed on Model 4, in which Li-ion diffusion was observed. Figure 4 compares the structural data obtained using the FF parameters with those obtained using FPMD. The FPMD-derived cell volume was 1049 Å^3^, which was in good agreement with the DFT-derived cell volume of 1048 Å^3^. Therefore, the obtained FF parameters are expected to enable simulations with the same level of accuracy as FPMD.

**Figure 5:**
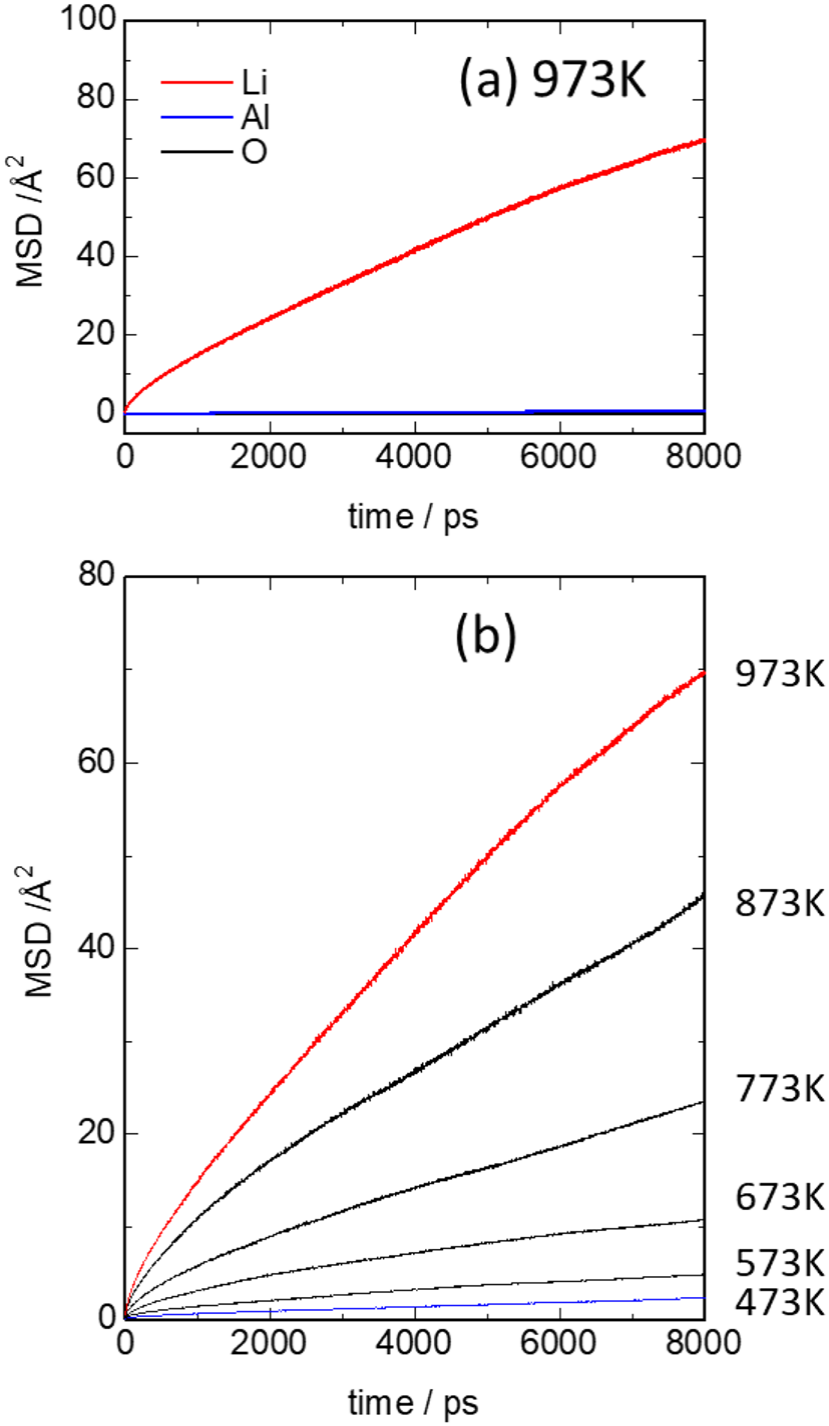
Mean square displacement (MSD) plots obtained by force field molecular dynamics (FFMD) calculations for Model 4, which contains a 4.5 mol% excess of Li ions and 1.5% deficiency (vacancies) of Al ions. (**a**) MSD plots of Li, Al, and O at 973 K. (**b**) MSD plots of Li ions at 473–973 K.

**Figure 6:**
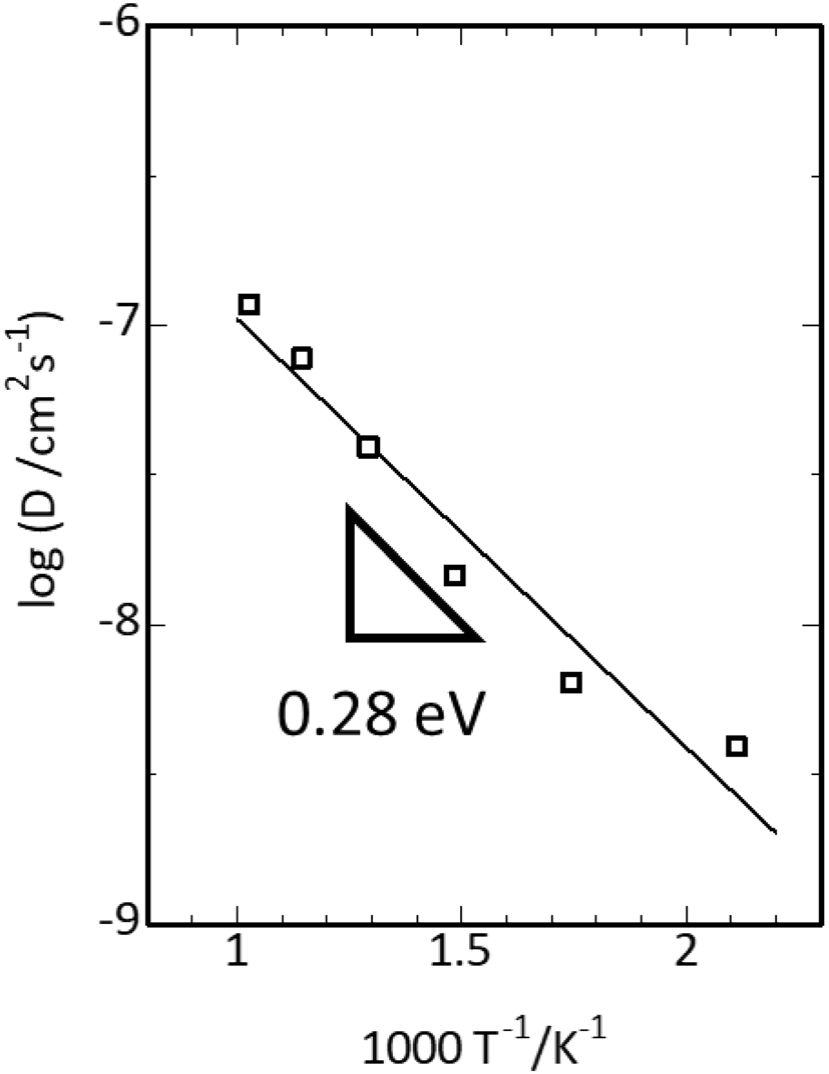
Arrhenius plot for the diffusion coefficient of Model 4 (4.5 mol% excess Li ions) by force field molecular dynamics (FFMD) calculations performed at 473–973 K. The activation energy evaluated from the straight-line slope is 0.28 eV.

High-throughput FF simulations were performed on a 4 × 4 × 4 supercell. First, the constant-ratio composition of LiAl_5_O_8_ (Model 1) was considered. The GA was performed for 714 generations to determine the most stable Li and Al arrays, which reproduced a spinel structure with Al ions occupying the tetrahedral and octahedral sites and Li ions occupying the octahedral sites. In contrast, the model with 3–5 mol% excess Li (Model 4) showed a rock-salt type structure with most of the Li ions occupying octahedral sites. Figure 5 shows the MSD at simulation temperatures of 473–973 K. The MSD of Li showed good linearity with respect to time, indicating that a quantitative evaluation was possible. We also performed the FFMD calculations for Model 1 and 2 with a 4 × 4 × 4 supercell at 973 K, and the MSD profiles are shown in Supplementary Fig. 1. According to the results of FPMD calculations, Li migration was not observed for both models (Fig. 3). The Arrhenius plot (log *D* vs 1/*T*; Fig. 6) shows good linearity, and the obtained activation energy is 0.28 eV. This result is in good agreement with the work of Mo et al.^23^, who obtained values of 0.11–0.33 eV by first-principles calculations using the nudged elastic band method^22^. The room-temperature diffusion coefficient was extrapolated from the straight line of the Arrhenius plot, and the room-temperature ionic conductivity, calculated based on the Nernst–Einstein equation, was 3.2 × 10^−6^ S/cm. cc; however, the calculated ionic conductivities were lower. We inferred that this was due to the low concentration of Li-ion carriers that can diffuse throughout the structure. Although defective LiAl_5_O_8_ (Model 4) exhibits lower ionic conductivity than that of Li_7_La_3_Zr_2_O_12_ for solid electrolytes, the calculated conductivity of 3.2 × 10^−6^ S/cm is sufficient for using it as coating material for Li metal. The overvoltage was estimated to be ~ 3 mV, considering the current density of 1 mA/g for the 100 nm thick coating material^12^. The Li-ion conduction properties of defective LiAl_5_O_8_ (Model 4) are comparable to or better than those of other candidate coating materials, including LiF (3 × 10^−9^ S/cm)^46,47^ and LiPON (~ 2 × 10^−6^ S/cm)^48^.

**Figure 7:**
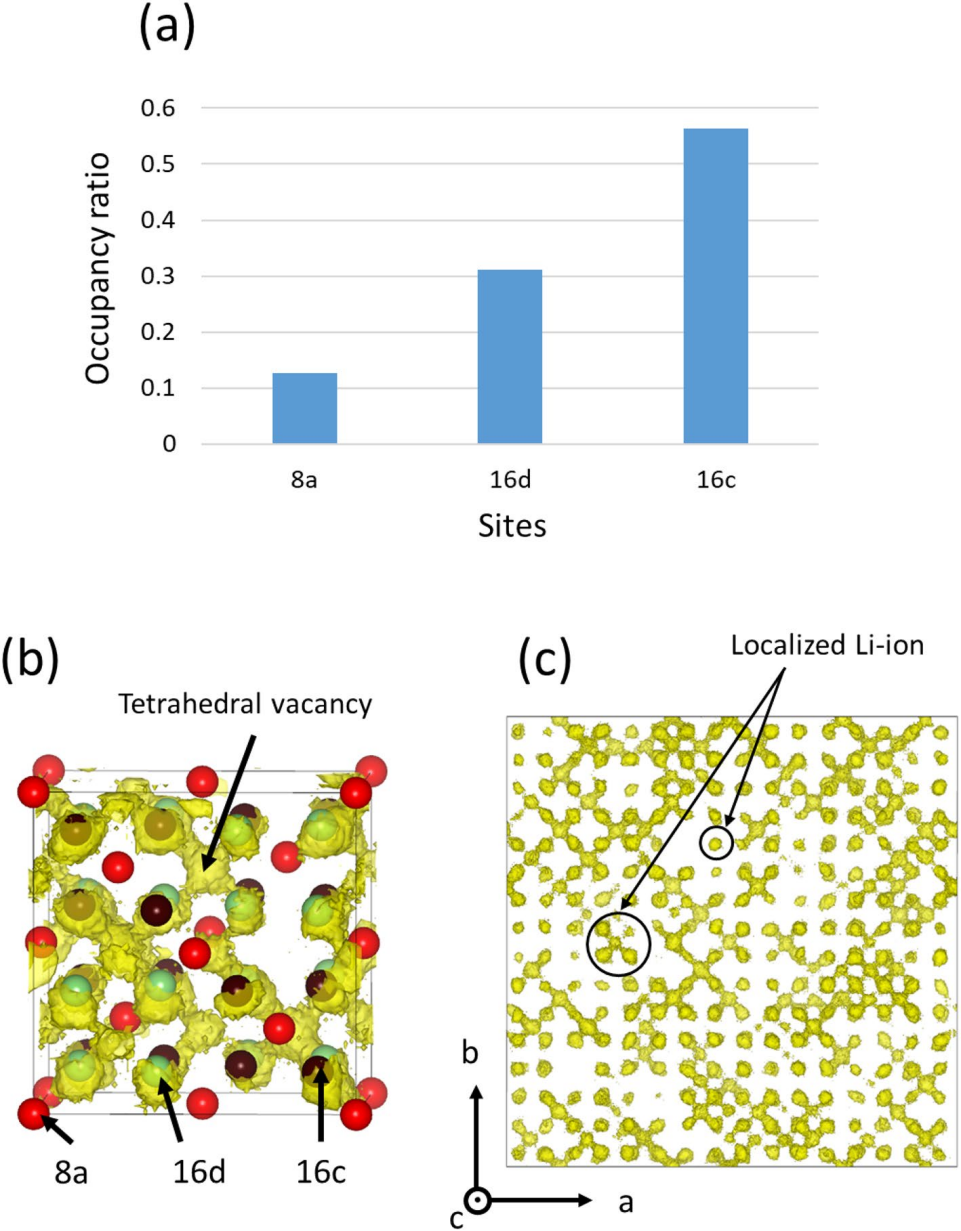
Li-ion array in Model 4 (4.5 mol% Li excess, 1.5 mol% Al deficiency) calculated by molecular dynamics (MD) at 973 K. (**a**) Occupancy ratio of Li ions among 8a, 16d, and 16c sites obtained by integration of Li population density within a radius of 1.2 Å centered at each site. Total occupancy ratio for 8a, 16d, and 16c sites set as unity. (**b**) Population density distribution of Li ions (yellow iso-surface) obtained by force field molecular dynamics (FFMD) calculations at 973 K for a 4 × 4 × 4 supercell folded into a 1 × 1 × 1 conventional spinel lattice. The red, green, and brown spheres represent 8a, 16d, and 16c sites, respectively. (**c**) Population density distribution of Li ions without folding of the supercell.

Figure 7a shows the site-occupancy ratio of Li ions in Model 4 (4.5 mol% Li excess, 1.5 mol% Al deficiency) during MD calculations at 973 K, and Fig. 7b,c show the population density distribution of Li ions in the lattice. The cation distribution in this structure does not show the cation ordering feature of *P*4_3_32; therefore, *Fd*$\overline{3 }$*m* symmetry is adopted hereinafter. The obtained results show that Li is mainly distributed at the octahedral 16d and 16c sites, because the tetrahedral 8a sites are occupied mainly by Al ions. The 16d sites are also partially occupied by Al ions; thus, Li ions preferentially occupy the vacant 16c sites when the structure contains excess Li ions (Fig. 7a).

For visibility purposes, a 4 × 4 × 4 supercell cell was folded into a 1 × 1 × 1 conventional spinel lattice, as shown in Fig. 7b. The 8a, 16d, and 16c cation sites are displayed as colored spheres, and the population density of Li ions is displayed as a yellow iso-surface. There was no substantial distribution of Li around the tetrahedral 8a sites owing to the presence of Al ions. Instead, the Li ions were mainly distributed around the 16d and 16c sites. Notably, the Li migration behavior is clearly visible between the octahedral 16c and 16d sites passing through the tetrahedral vacancy sites, as shown in Fig. 7c. The tetrahedral vacancy sites correspond to the 48f crystallographic sites. This migration trajectory agrees with the knock-off mechanism proposed by Mo et al.^23^, wherein Li ions do not hop linearly but instead traverse via tetrahedral vacancy sites. Figure 7c shows the population densities of Li ions in the 4 × 4 × 4 supercell. The population density distribution formed a hopping pathway between both edges of the supercell model, indicating that long-range Li-ion migration is allowed in the structure. However, some Li ions were disconnected from the pathway because they were surrounded by immobile Al ions. These localized Li ions do not contribute to the net ionic conductivity. Therefore, the octahedral 16d and 16c sites constitute the main diffusion path. However, the conduction path is limited owing to the presence of Al ions. This could be the reason why, despite its low activation energy, this structure exhibited a conductivity of only approximately 10^−6^ S/cm at room temperature.

## Conclusion

In this study, the mechanisms of defect formation and Li-ion conduction in spinel-type LiAl_5_O_8_ as a coating material for Li-metal anodes were investigated using FPMD, FFMD, and GA calculations. First-principles calculations indicated that stoichiometric LiAl_5_O_8_ does not have Li-ion conductivity. However, a model with Li excess and Al vacancies (Li_1.45_Al_4.85_O_8_) revealed that the formation of interstitial Li-ion sites was both thermodynamically conceivable and conducive to Li-ion diffusion. The Li-ion conductivity was quantitatively evaluated by FFMD. The calculated activation energy was 0.28 eV, which is comparable to that of garnet-type Li_7_La_3_Zr_2_O_12_, a highly Li-ion conductive material (0.33 eV, based on a previous report using FPMD calculation^45^, and 0.27 eV, according to the results of the FFMD calculation in this study, as shown in Supplementary Fig. 2).

In contrast, the extrapolated room-temperature ionic conductivity was on the order of 10^−6^ S/cm and did not reach the high room-temperature Li ionic conductivity of 3 × 10^−4^ S/cm obtained for Li_7_La_3_Zr_2_O_12_^49^. The visualization of diffusion pathways by MD calculations indicated a decrease in conductivity because of the limited diffusion pathways between the octahedral 16d and 16c sites via tetrahedral vacancy sites. However, as the layer thickness will be several tens to several hundreds of nanometers for coating purposes, the present Li-ion conductivity is sufficient for practical usage. Furthermore, the calculated low activation energy is highly beneficial for application in low-temperature environments because the decrease in conductivity is small. Contrastingly, pin-hole creation or fracture of coating materials, owning to the brittleness of ceramics and nano-sized thin film layers, is required to realize practical usage in the future.

## Supplementary Information

Supplementary Figures.

## Data Availability

The data that support the findings of this study are available from the corresponding author upon reasonable request.
