# Supplementary material for: Computational studies on defect chemistry and Li-ion conductivity of spinel-type LiAl5O8 as coating material for Li-metal electrode
Source: Sci Rep. 2022 Oct 5;12:16672. doi: 10.1038/s41598-022-20289-2 (PMC9534845; doi:10.1038/s41598-022-20289-2)
Supplement: Supplementary file 1 — Supplementary Figures. [file 41598_2022_20289_MOESM1_ESM.docx]

**Supplementary Information**

Computational Studies on Defect Chemistry and Li-Ion Conductivity of Spinel-Type LiAl_5_O_8_ as Coating Material for Li Metal Electrode

Shuntaro Miyakawa^1)^, Shogo Matsuda^2)^, Naoto Tanibata^2)^, Hayami Takeda^2)^, Masanobu Nakayama^2)*^, Takaya Saito^1)^, Svetlana Fukuchi^1)^

1. Advanced Battery Research Office, Research Institute of Advanced Technology,

SoftBank Corporation, Kaigan, Minato-Ku, Tokyo 105-7529, Japan

1. Department of Advanced Ceramics, Nagoya Institute of Technology, Goiso, Showa-ku, Nagoya, Aichi 466-8555, Japan

* E-mail: masanobu@nitech.ac.jp


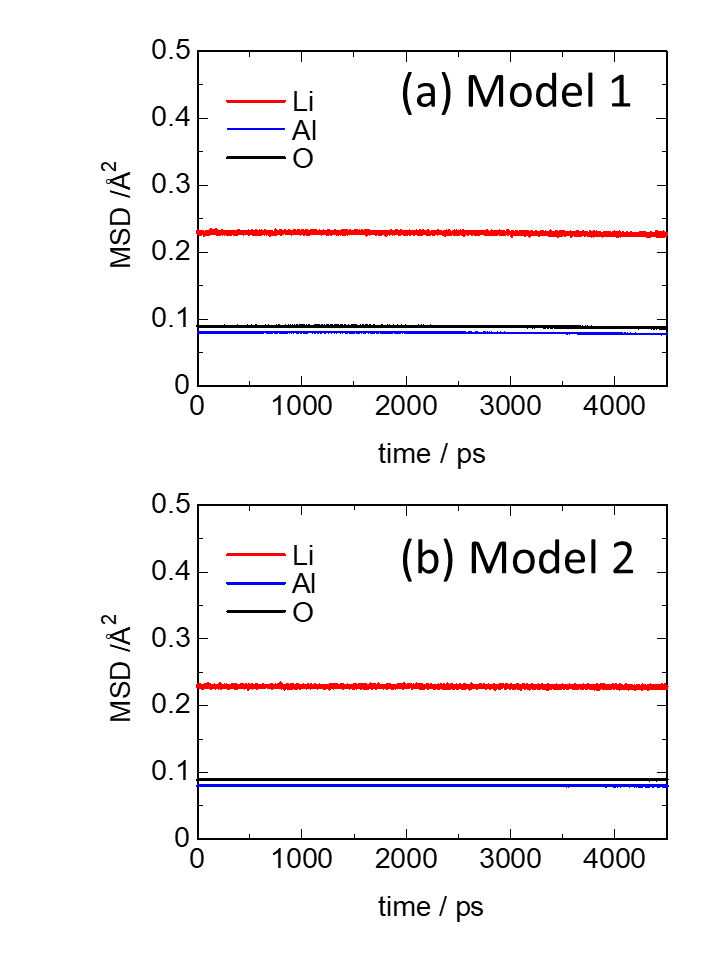


**Supplementary Figure 1** Mean square displacement (MSD) plots obtained using force field molecular dynamics (FFMD) calculations at 973 K for (a) Model 1 and (b) Model 2, which contains 4.5 mol% vacancies at Li sites.


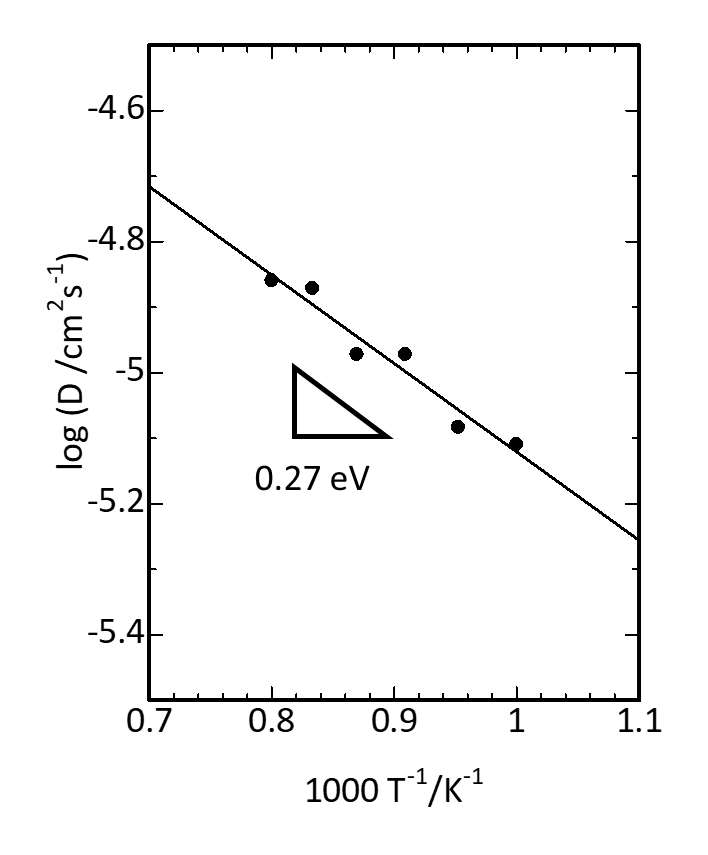


**Supplementary Figure 2** Arrhenius plot for the diffusion coefficient of Garnet-type Li_7_La_3_Zr_2_O_12_ by force field molecular dynamics (FFMD) calculations using Cuckoo-search-optimized FF parameters as described in the main text. The MD simulations were performed at 1000–1200 K. The activation energy evaluated from the straight-line slope is 0.27 eV.
